# Two Cases of Loss of Power over the Voluntary Muscles

**Published:** 1819-01-01

**Authors:** Richard Moulson


### *Two Cases of Loss of Power over the Voluntary Muscles.*

By Richard Moulson, M. D.

E. H. ætat. 25, I saw for the first time on the 24th of September, 1817, labouring under a loss of voluntary power over the muscles of the extremities. This woman, ten years ago, had a venereal sore throat, for which she was treated accordingly; she had also been in St. Bartholomew's Hospital, under the care of one of the physicians, where, from what I could learn, she had taken mercurials with decoctum sarsaparillæ. When first I saw her, she was confined to bed, unable to assist in feeding herself, or turning from any position in which she might be placed; and in this state she continued thirteen months. The intestines and bladder had participated so much in the disease, that purgatives, however strong they were given to her, seemed to have no effect whatever, unless assisted by clysters; these were regularly administered every day for several months, and her water was drawn off by the catheter night and morning. Her pulse and appetite, considering the situation she was in, were very good. The catamenia had not appeared for twelve months previous to my seeing her, nor have they at the time I am now writing. Even in the depth of winter this poor woman was bathed in sweat; which, in a great measure, will account for some of the principle viscera retaining their healthy structure. What she generally complained of was a great oppression within the bowels, for want of a free passage, and a pain at the back of the head and neck. So deplorable a case did this at first sight appear, that little hopes were entertained of an amendment; yet, as there was life, it was necessary that it should be made as comfortable as possible. Upon examining the neck, an exostosis of the second vertebra was discovered, which seemed the chief cause of her complaints; for nothing else could be found to account for her melancholy situation. A seton was immediately placed close to this exostosis, and it was regularly dressed with the unguentum hydrargyri fortius. Purgatives were regularly administered, and in order to support her strength various tonics were prescribed for her; but these did not seem to do her any good; on the contrary, they oppressed her stomach and bowels, so that, during the major part of her illness, she took nothing but her ordinary food and purgatives.

In March, 1818, she was able to move her arms and legs; and with the assistance of another woman, to get out of bed, and sit up for a short time in an arm chair. In the beginning of April, with the assistance of crutches, she could move about the house, when I thought the waters at Buxton might be of service to her. Thither she went, and remained six weeks; and on her return home, she was able to walk about with the aid of a stick. At present, although much debilitated, she walks without any assistance; enjoys a tolerable appetite; has no necessity for opening medicines nor catheter; and is, in fact, in comparatively good health. Her right arm is still unwieldy, and the index and middle fingers of that hand are still very stiff. As might have been expected, she is much emaciated; yet, by proper care and attention, she may probably become plump again. Whilst at Buxton, she suffered the part where the seton was inserted to heal up. The seton, however, must be renewed, as she tells me that occasionally she has pains in her head which, at the time, affect her memory: she also thinks, that since the seton has been removed the bone has again enlarged. To appearance, this woman has now nothing the matter with her, and would in no way attract the attention of a passer-by, further than the remark, that she was a poor looking wretch. There seems, however, to be a disposition to exostosis in several bones of the thorax, which will require great attention; how far medicinal treatment may be able to arrest the disease, I am at present unable to determine. Should death admit of an investigation, or medicine effect a cure, I may probably refer to this case at a future period.

The above relation shows what may be done under the most unfavourable circumstances; and if the mention of it will only stimulate others to perseverance in a similar case, I shall consider myself amply repaid for transcribing it.

Mrs. F. ætat. 36, I saw for the first time on the 13th of February, 1818, labouring under a loss of voluntary power over the muscles of the inferior extremities. Sixteen months previous to my seeing her she had been confined, and from that time until I visited her she never had her clothes on, nor was she able to get out of bed to have it made, unless assisted by the persons around her. The use of the superior extremities she had at perfect command, so that being propped up with pillows, she was enabled for a short time in the day to sew for her family. But this she could not long continue, owing to a pain in her back, which tormented her if not in a recumbent posture. Laxatives were the only medicines she had taken before I saw her, and these had every now and then been required in her inactive state of life. Her pulse was weak ; tongue a little furred from a disordered stomach she had experienced from the time of her confinement; catamenia regular, except in being more profuse than usual ; bowels variable, more generally constipated than otherwise ; her inferior extremities had become much emaciated. What particularly attracted my attention were the pains in her back and down the thighs, which were very great. Upon examining the spine, there seemed to be a slight protuberance over the lumbar region, where it was decided that caustic issues should be placed. On examining per vaginam, a procidentia uteri was discovered, which immediately was supported by a pessary ; and the following medicines were prescribed :

℞. Pilulæ Hydrargyri ; rhei rad. pulv. āā ʒj ; mucil. g. acaciæ q. s. ut ft. ; pilulæ xxiv quarum sumat. ij, omni alterna nocte.

℞. Infusi strychnos nucis vomicæ, aquæ puræ āā ℥iv ; tinct. cardamomi com. ℥ß, M. ft. mistura, cujus sumat. ℥j ter de die.

The pills she took until her bowels became natural, when she discontinued them. On the 7th of March, more of the infusion and less of the water composed the mixture. On the 4th of April, not perceiving any particular good effects from the mixture, more than that it agreed very well with the patient, and strengthened her appetite, I was induced to order the pil. ferri cum myrrhâ, with a bark mixture, to recruit her debilitated frame. These, however, upon the 29th of April I discontinued, finding that they did not agree with her so well as the infusion of the nux vomica, to which I again had recourse. On the 20th of May, finding that her bowels were disordered and her appetite began to flag, I ordered her the following powder, to be taken every day an hour before dinner.

℞. Rhei rad. pulv. gr. v ; magnesiæ ℈ß ; zingiber. pulv. gr. iij, M. ft. pulvis. This powder, in conjunction with the nux vomica, she took until the 8th of August, when she was discharged cured.

It is impossible to describe the feelings of this poor woman, after being bed-ridden so long ; she now walks about the town with comfort and ease, and enjoys very good health. The pessary is still continued, to support the uterus; but every night it is removed, and the following morning replaced. The part where the caustic issues were placed is now perfectly healed. What effect the administration of the nux vomica had in removing the complaint, I really am unable to say. From the experiments of Drs. Magendie, Delile, Desportes, and Orfila, who found that the nux vomica exercised a peculiar action upon the spinal marrow, and from the practice of Dr Fouquier, in cases of paralysis, I have no doubt, that if a fair and impartial trial be given to this substance, and its powers honestly stated, and not eulogized, it will be found to be a valuable acquisition to our present stock of remedies. As I never met with an extract of the nux vomicæ, and have only used an infusion,* I have never seen those "tressaillemens soudains et passagers" which have been observed by others. Plenck, in his Toxicologia, makes mention of a tincture. Alibert states that the alcoholic extract is the most certain, and at the same time the most energetic preparation. I have administered the infusion in several dyspeptic cases with advantage, even after other more likely remedies had failed; in other cases, I have discontinued it from not seeing any good derived from it. In some cases of gastrodynia, it has proved immediately serviceable, after the oxide of bismuth had failed; and vice versâ.

The observation of Lieutaud "Que la moëlle de l'épine étoit le siége des convulsions qui laissoient la liberté des sens et celle de la parole," I believe to be applicable to many cases of paralysis. In Mrs. F's case, the procidentia uteri was the primary cause of her complaints, and no doubt it pressed upon the nerves distributed to the extremities, and so occasioned the paralysis;† yet, after the malady had continued for so long a time, I query if the supporting of the uterus would alone have regained her the use of her limbs. For the benefit of those who may not have met with the following observation of Alexander Trallianus, I shall take the liberty of transcribing it, as an at-

---

* A drachm of the nux vomica, reduced as fine as possible, was infused in a pint of boiling water, and when cooled, strained.

† Morgagni quotes Saxonia having observed the superior and inferior extremity of one side affected with a tremor and a stupor, from an inclination of the uterus to that side. De Sedibus et Causis Morborum, Epist. xlviii. Art. 33.—Lameness from a slight displacement of the uterus in irritable subjects is a much more common occurrence than is generally supposed; and it is well known, that a stricture of the urethra in some men will induce a most serious disturbance in the system, occasioning pains analogous to rheumatism and gout, and which will defy all medicinal treatment until the stricture be removed.

tention to it will undoubtedly lead to more correct ideas of spasmodic and paralytic affections than what are generally entertained. "Si igitur ex superioribus partibus quædam affectæ fuerint, nempe oculus, nasus, aut lingua, aut quædam in facie, constat, quod ipsum cerebrum habeat morbum, illique primario succurrendum sit. Si ergo nulla ex prædictis partibus sensu aut motu aut utroque læsa fuerit, necesse est spinalem medullam laborare, aut aliquem nervorum ex ipsa prodeuntium affectum esse statuere. Attendito igitur deligenter, quæ sit pars affecta, et unde initium trahit, aut a qua vertebra id aut nervo recipiat, atque illi curationem adhibeto: non autem ut vulgo, symptomatibus tantum obsistito."

*Halifax, Yorkshire, Sept.* 21, 1818.

